# 17β-Estradiol Is Required for the Sexually Dimorphic Effects of Repeated Binge-Pattern Alcohol Exposure on the HPA Axis during Adolescence

**DOI:** 10.1371/journal.pone.0032263

**Published:** 2012-02-22

**Authors:** Magdalena M. Przybycien-Szymanska, Roberta A. Gillespie, Toni R. Pak

**Affiliations:** Department of Cell and Molecular Physiology, Loyola University Chicago, Stritch School of Medicine, Maywood, Illinois, United States of America; University of Florida, United States of America

## Abstract

Alcohol consumption during adolescence has long-term sexually dimorphic effects on anxiety behavior and mood disorders. We have previously shown that repeated binge-pattern alcohol exposure increased the expression of two critical central regulators of stress and anxiety, corticotrophin-releasing hormone (CRH) and arginine vasopressin (AVP), in adolescent male rats. By contrast, there was no effect of alcohol on these same genes in adolescent females. Therefore, we tested the hypothesis that 17β-estradiol (E_2_), the predominant sex steroid hormone in females, prevents alcohol-induced changes in CRH and AVP gene expression in the paraventricular nucleus (PVN) of the hypothalamus. To test this hypothesis, postnatal day (PND) 26 females were ovariectomized and given E_2_ replacement or cholesterol as a control. Next, they were given an alcohol exposure paradigm of 1) saline alone, 2) acute (single dose) or 3) a repeated binge-pattern. Our results showed that acute and repeated binge-pattern alcohol treatment increased plasma ACTH and CORT levels in both E_2_- and Ch-treated groups, however habituation to repeated binge-pattern alcohol exposure was evident only in E_2_-treated animals. Further, repeated binge-pattern alcohol exposure significantly decreased CRH and AVP mRNA in Ch-, but not E_2_-treated animals, which was consistent with our previous observations in gonad intact females. We further tested the effects of E_2_ and alcohol treatment on the activity of the wild type CRH promoter in a PVN-derived neuronal cell line. Alcohol increased CRH promoter activity in these cells and concomitant treatment with E_2_ completely abolished the effect. Together our data suggest that E_2_ regulates the reactivity of the HPA axis to a repeated stressor through modulation of the habituation response and further serves to maintain normal steady state mRNA levels of CRH and AVP in the PVN in response to a repeated alcohol stressor.

## Introduction

Sexually dimorphic patterns of addictive behavior emerge during adolescence and often persist in adulthood. For example, in early adolescence (ages 12–17), the amount and frequency of alcohol consumption does not differ between boys and girls, yet it becomes markedly skewed in young adults with males consuming significantly more alcohol than females [1]. Consumption of alcohol for individuals between the ages of 18 and 25 tends to be excessive and sporadic (“binge” drinking) which leads to increased risk-taking behavior [1], [2], [3], [4], [5]. One possible explanation for these sex differences is that early experimentation with alcohol (i.e. 12–16 years old) differentially activates the stress response, and/or centrally-mediated reward systems, resulting in divergent behavioral patterns that emerge in late adolescents/young adults.

Correlative studies have demonstrated that over 50% of patients with alcohol dependency also have anxiety-related or depression-related psychiatric disorders [6], and these types of disorders are often associated with an abnormal stress response. Under normal physiological conditions, an acute psychological or physical stressor activates the hypothalamo-pituitary adrenal (HPA) axis. Hypothalamic corticotrophin-releasing hormone (CRH) stimulates adrenocorticotropic hormone (ACTH) release from the anterior pituitary which in turn, causes the release of adrenal glucocorticoids. This sequence of events sets up a negative feedback system whereby increased circulating glucocorticoid levels serve to inhibit the additional release of hypothalamic CRH. Interestingly, alcoholic women have a higher incidence of clinically diagnosed anxiety disorders compared to alcoholic men [7], [8], possibly due to the inherent sex differences in the stress responses of the HPA axis [9], [10].

Previously, we showed that adolescent repeated binge-pattern alcohol exposure altered the central expression of CRH and AVP mRNA in the paraventricular nucleus (PVN), the major brain region involved in modulating the physiological stress response [11]. Importantly, the effects of alcohol on these central gene expression profiles in the PVN occurred only in males, yet the peripheral alcohol-induced CORT response was similar in both sexes [11]. Overall, these data demonstrated that repeated binge-pattern alcohol exposure during puberty fundamentally dysregulated the HPA axis in a sex-specific manner, yet the molecular mechanisms mediating this sexual dimorphism remain unknown.

Puberty is demarcated by dramatic changes in circulating gonadal steroid hormone levels and increased gonadal growth, therefore it is reasonable to predict that the sexual dimorphism previously observed is regulated, in part, by differences in circulating gonadal steroid hormones. The main circulating gonadal steroid hormone in reproductively competent females is 17β-estradiol (E_2_). Therefore, we hypothesized that the failure of binge-pattern alcohol exposure to increase CRH and AVP gene expression in the PVN of female rats is due to the presence of circulating E_2_. We also tested the effects of concomitant alcohol and E_2_ on CRH promoter activity in a PVN-derived cell line. Overall, our results indicated that E_2_ was required for very specific aspects of the alcohol-mediated changes in the female HPA axis, but that other, as yet undetermined, factors are also important for mediating the overall sexually dimorphic response of the HPA axis to alcohol.

## Materials and Methods

### Ethics Statement

All animal procedures were approved by the Loyola University Stritch School of Medicine Institutional Animal Care and Use Committee (IACUC) permit #2007027.

### Animals

Female Wistar rats were purchased from Charles River Laboratories (Wilmington, MA) at weaning (postnatal day (PND) 23) and allowed to acclimate for 3 days after arrival. On PND 26, they were bilaterally ovariectomized (OVX) and implanted with a 0.5 cm silastic capsule containing either crystalline 17β-estradiol (E_2_) or cholesterol (Ch). This dose has been shown in previous studies to yield plasma E_2_ levels of approximately 64 pg/ml [12], [13], which is consistent with levels observed during diestrous [14] and was chosed to mimic low circulating hormone levels present in peripubertal females. Animals were allowed to recover from surgery until PND 30 at which time they were handled for 5 min/once/day for 7 days to acclimate the animals to non-specific handling stress. EtOH treatment (see details below) began on PND 37 which has been defined in the literature as peri-puberty in rats [15], [16], [17]. Animals were housed in pairs on a 12∶12 light/dark cycle with lights on at 07.00 h and food and water were available *ad libitum* for the duration of the treatment paradigm. All procedures were approved by the Loyola University Stritch School of Medicine Institutional Animal Care and Use Committee.

### Repeated Binge-Pattern Alcohol Exposure Paradigm and Treatment Design

E_2_-treated and Ch-treated animals were randomly assigned to one of three groups: 1) saline treated (N = 8/group), 2) acute EtOH treated (N = 8/group), or 3) repeated binge-pattern EtOH treated (N = 8/group, 3 g/kg/day). The animals in the repeated binge-pattern EtOH group received one intraperitoneal (IP) 3 g/kg (20% v/v in saline) EtOH injection every morning at 10:00 AM for 3 consecutive days, followed by 2 days of saline injections, and then injected for additional 3 days with EtOH. This repeated binge-pattern EtOH exposure paradigm has been used previously to mimic the pattern of binge alcohol consumption in adolescents [11], [18], [19] and it has been shown that in pubertal animals this IP route of alcohol administration does not yield significantly different blood alcohol concentrations (BAC) or CORT levels compared to oral gavage [20] (Pak, unpublished data). The animals were given alcohol in the morning (10:00 AM, CST) and previous studies showed that morning alcohol administration does not interfere with normal feeding behavior and does not result in body weight differences between alcohol-treated and control animals [11], [18]. The acute EtOH-treated group was given 7 days of saline IP injections (0.9% once/day) followed by one 3 g/kg of EtOH IP injection on the last day of treatment (day 8). The saline group was treated with saline alone (once/day) for 8 consecutive days. On the last day of treatment (PND 44), animals were killed by rapid decapitation 1.0 h following the final injection.

### Blood alcohol concentration (BAC) assay

Blood alcohol levels were determined by measuring the change in absorbance at 340 nm following enzymatic oxidation of EtOH to acetylaldehyde (Point Scientific Alcohol Reagent Kit). Assay range is 0 to 400 mg/dl and intra and interassay CV = 7.86 and 8.9%, respectively.

### Plasma hormone measurements

Plasma ACTH and CORT were measured using radioimmunoassay (RIA) as previously reported [11], [19]. Briefly, ^125^I-ACTH (DIASORIN, Stillwater, MN), rabbit IgG-ACTH-I primary antibody (Ig Corp, Nashville, TN) and goat anti-rabbit G-globulin secondary antibody (CalBiochem, San Diego, CA) was used for measurements of ACTH. ^3^H-CORT (PerkinElmer, Waltham, MA), rabbit CORT antiserum (MP Biomedicals, Solon, OH), and CORT-standards (4-PREGNEN, 11b, 21-DIOL-3,20-DIONE-Steraloids, Inc) were used to measure CORT. Intraassay CVs were 4.94 and 4.96%, and interassay CVs were 14.9 and 9.7%, respectively.

Circulating E_2_ levels were measured by E_2_ Extraction-Chromatography-RIA (Endocrine Technology and Support Laboratory, Oregon National Primate Center, Oregon Health Science University, Portland, OR). The reported intraassay and interassay CV were 12% and 18%, respectively.

### Tissue collection and qRT-PCR

Animals were killed by rapid decapitation 60 min following the last injection. Trunk blood and brains were collected and brains were rapidly frozen in isopentane (−30°C) and stored at −80°C. Brain samples and qRT-PCR were performed as previously reported. Briefly, brains were sectioned at 200 µm on a freezing microtome and the paraventricular (PVN) and supraoptic (SON) nuclei were microdissected using a 0.75 mm Palkovit's brainpunch tool (Stoelting Co., Wood Dale, IL). The specificity of the microdissected regions was confirmed using The Rat Brain in Stereotaxic Coordinates, Fourth Edition Atlas (G. Paxinos and C. Watson). For the PVN, a 0.75 mm area was microdissected on each side of the third ventricle between 0.8 mm and 2.12 mm posterior to Bregma, 8 mm below the top of the brain [21]. For the SON a 0.4 mm area was microdissected 9.5 mm below the top of the brain between 0.8 mm and 3.14 mm posterior to Bregma. Total RNA was isolated using Trizol reagent (Invitrogen Inc., Carlsbad, CA) according to the manufacturer's directions. Following RNA isolation, 0.5 µg total RNA was reverse transcribed using the First Strand Synthesis SuperMix for qRT-PCR (Invitrogen Inc., Carlsbad, CA). Roche FastStart SYBR Green Master Mix was added to intron-spanning AVP specific upper and lower primers: 5-GGGCAGGTAGTTCTCCTCCT; 5-CACCTCTGCCTGCTACTTCC) and intron-spanning CRH primers: 5-GAGAAAGGGGAAAGGCAAAG; 5-ATCAGAATCGGCTGAGGTTG). Quantification of the target gene expression was achieved by extrapolating from standard curve of known concentrations of AVP or CRH ran simultaneously in the same plate. All samples were normalized to the hypoxanthine guanine phosphoribosyl transferase 1 (HPRT) housekeeping gene, as it is not altered by EtOH treatment [11].

### Cell Culture

The rat PVN-derived cell line (IVB cell line, generously provided by Dr. John Kaskow, University of Cincinnati) was used for all transient transfections. Cells were maintained in DMEM containing 4.5% glucose and L-glutamine (HyClone Laboratories, Logan, UT) supplemented with 10% fetal bovine serum (FBS). Cells were grown to 90% confluency and all transient transfections were performed within the same 10 passages.

### Reporter gene constructs and expression vectors

The full-length CRH promoter was generously provided and validated by Dr. Audrey F. Seasholtz (University of Michigan, Ann Arbor, MI) and then modified as follows: the full-length promoter fragment (−2125/+94) was excised from the pUC18 vector by restriction enzyme digestion for EcoR1 (5′) and HINDIII (3′) and subsequently subcloned into the promoterless luciferase vector (pGL3 basic, Promega Corp., Madison, WI). The pRL-tk luciferase reporter vector (Promega Corp., Madison, WI) was used as an internal control for calculating plasmid transfection efficiency.

### Transient Transfections and Dual Luciferase Assay

Cells were plated at a density of 20,000 cells/well in opaque 96-well plates for 24.0 h prior to transfection to achieve a final confluency of 80–90%. All constructs were transfected in replicates of six wells within each assay, and each transfection assay was repeated in a minimum of 6 independent experiments. Transfections were performed using a lipid-mediated transfection reagent (Fugene6, Roche Molecular Biomedical, Indianapolis, IN) according to the manufacturer's instructions. 8 h after transfection cells were incubated in vehicle, 10 nM E_2_ or an estrogen receptor-beta (ERβ)-specific agonist, 100 nM 5alpha-androstane-3beta,17beta-diol (3βdiol). Cells were incubated with hormones overnight (15.0 h) and then treated with 100 mM EtOH for 2.0 h. The Dual Luciferase Reporter (DLR) kit (Promega Inc., Madison, WI) was used according to manufacturer's directions. Briefly, cells were lysed in 20 µl of lysis buffer, incubated on a shaker for 20 min at room temperature and then loaded into a multiple well plate reader (Synergy HT, Biotech). The plate reader is equipped with dual injectors and automatically dispensed 100 µl firefly luciferase substrate (LARII) followed by “stop-and glo” substrate for renilla luciferase. Results were analyzed using Gen5 software system (Biotech Inc., Winooski, VT).

### Statistical Analysis

Statistical analyses were performed by the Biostatistics Core Facility at Loyola University Stritch School of Medicine in consultation with Dr. James Sinacore. A 2×2 two-way analysis of variance (ANOVA) was used to test for interactions between hormone and alcohol treatments and for main effects of these treatments with respect to the following dependent variables: plasma BAC, plasma CORT levels, CRH mRNA in the PVN, and AVP mRNA in the PVN and SON. Tukeys post hoc test was used if ANOVA achieved significance. For *in vitro* studies, 2×3 two-way ANOVA was used to test for interactions between alcohol and specific hormone (vehicle, E_2_, 3βdiol) treatments and for main effects of vehicle or alcohol treatment with respect to the CRH promoter activity. Tukeys post hoc test was used if ANOVA achieved significance. All tests were performed using SigmaStat Statistical Analysis Software. A p-value of less than 0.05 was designated as statistically significant.

## Results

### Effects of Ch-, E_2_, and EtOH treatment on body weight in OVX adolescent female rats

**Figure 1:**
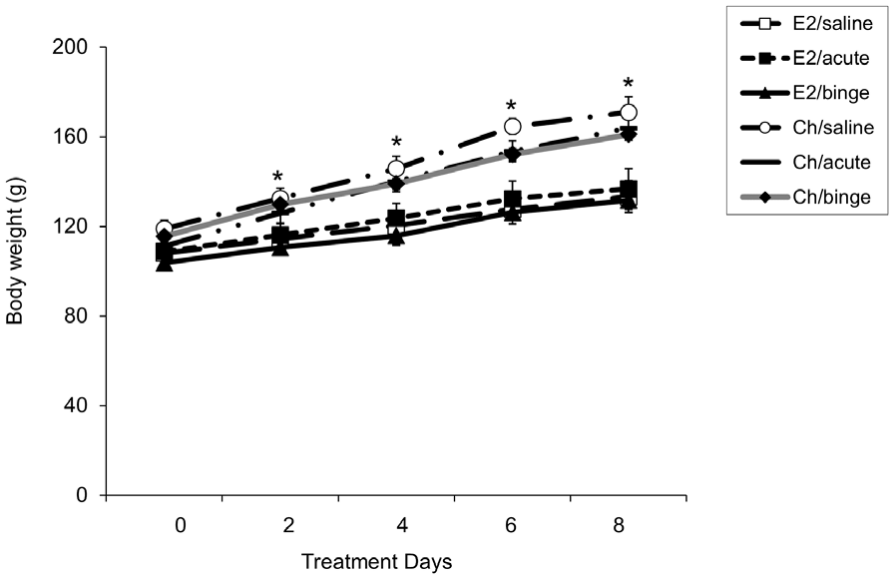
Effects of EtOH treatment on body weight of ovariectomized females replaced with Ch or E_2_ during adolescence. Mean body weights of animals in all treatments groups in females treated with daily IP injections of saline, acute EtOH, or repeated binge-pattern EtOH paradigm. Data are expressed as mean ± SEM. (_*_) indicates a statistically significant difference from control (p<0.05).

Animals were weighed every-other day during the course of the treatment paradigm in order to determine the effects of hormone and concomitant EtOH treatment on body weight. A repeated measures mixed ANOVA revealed that there was a significant main effect of age (F(1,44) = 9.9. p = 0.003), but not EtOH treatment, on body weight (F(2,44) = 0.252; p = 0.388). The Ch-treated animals gained weight at a faster pace compared to the animals treated with E_2_ (Fig. 1), which is consistent with the reported anorexigenic effects of E_2_. Concomitant EtOH treatment had no additional effects on body weight and all variation was exclusively due to hormone treatment (Fig. 1).

Circulating E_2_ levels were 65±7.3 pg/ml in E_2_-treated OVX females and were below the detectable limits of the assay in OVX Ch-treated females. Importantly, EtOH treatment did not alter E_2_ levels, as there were no differences in plasma E_2_ levels between EtOH treatment groups. These data, together with body weight data, indicate that E_2_ replacement via silastic capsules was effective in these experiments and raised plasma E_2_ levels to expected values.

### Blood alcohol concentrations (BAC) in Ch- and E_2_-treated OVX adolescent female rats

**Figure 2:**
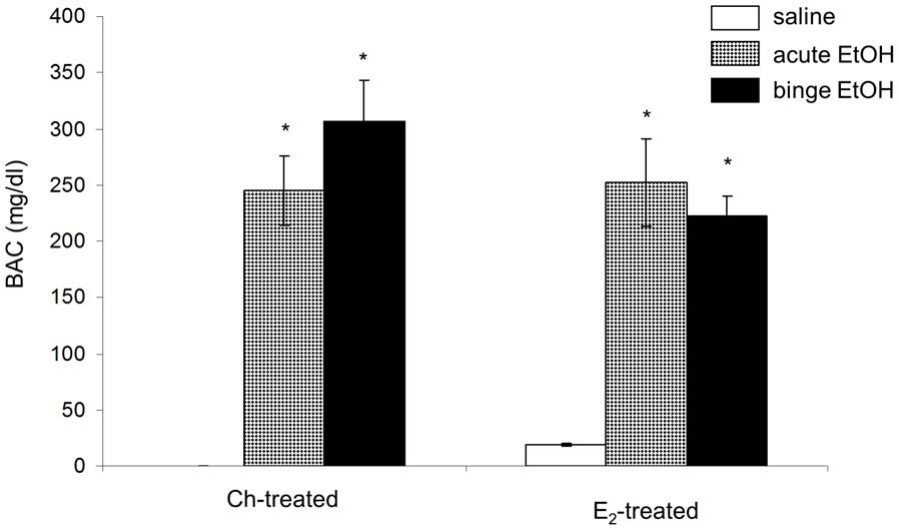
Effects of EtOH treatment on blood alcohol levels in ovariectomized female rats. Blood alcohol concentrations (BAC) 1.0 h post-injection in ovariectomized female rats given subcutaneous implants of Ch or E_2_ and then treated with saline, acute EtOH or repeated binge-pattern EtOH. Data are expressed as Data are expressed as mean ± SEM of EtOH mg/dl. (_*_) indicates a statistically significant difference from saline-treated controls (P<0.05).

Blood alcohol concentrations (BAC) were measured on the final day of treatment (day 8) 60 min following injections. EtOH treatment resulted in a BAC of 257.09±19.97 mg/dl and 275.77±22.94 in E_2_- and Ch-treated females, respectively (Fig. 2). These values are consistent with the defined BAC threshold of binge drinking [22]. Two-way ANOVA showed that there was a main effect of EtOH treatment (F(2,38) = 60.368, p<0.001) on BAC, but no main effect of hormone treatment (F(2,38) = 1.595; p = 0.516). BAC were similar in all groups regardless of hormone treatment.

### Effects of repeated binge-pattern EtOH exposure on plasma ACTH and CORT levels in Ch- or E_2_ – treated OVX adolescent female rats

**Figure 3:**
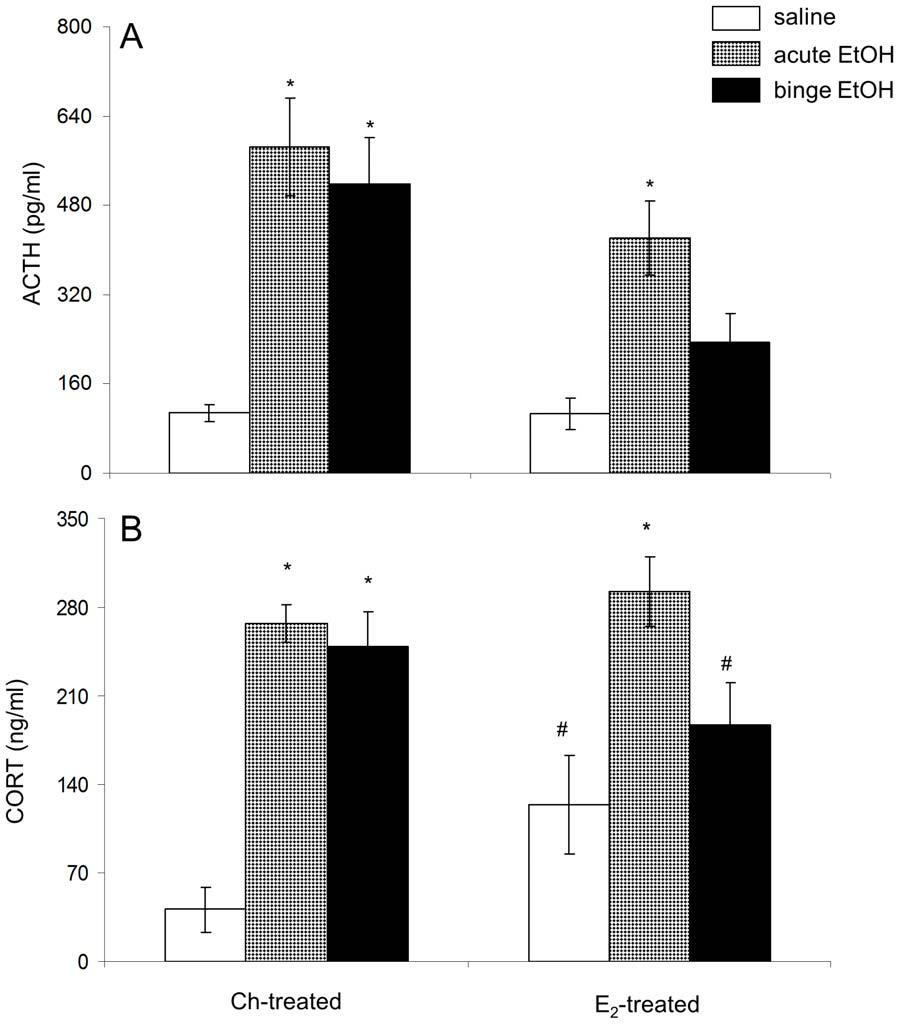
Effects of EtOH treatment on plasma ACTH and CORT levels in ovariectomized female rats replaced with Ch or E_2_. (A)Plasma ACTH and (B) CORT levels in ovariectomized female rats given subcutaneous implants of Ch or E_2_ and then treated with saline, acute EtOH or repeated binge-pattern EtOH. Data are expressed as mean ± SEM of ACTH and CORT ng/ml of blood. Dissimilar symbols indicate a statistically significant difference between groups (P<0.05).

Circulating ACTH and CORT are important peripheral indicators of a physiological stress response. Therefore, plasma ACTH and CORT levels were measured by RIA in Ch- and E_2_-treated animals in order to determine if E_2_ modulates the physiological stress response to EtOH. Overall, a two-way ANOVA revealed that there was a main effect of hormone treatment (F(1,35) = 10.563; p = 0.003), a main effect of EtOH treatment (F(2,35) = 24.219; p<0.001), and a significant interaction on plasma ACTH levels (Fig. 3A). Analysis of plasma CORT levels revealed that there was no main effect of hormone treatment (F(2,36) = 0.318; p = 0.73) and that there was a main effect of EtOH treatment (F(2,36) = 27.834; p<0.001) on the plasma CORT levels (Fig. 3B). There was also a significant interaction between the two factors (p<0.05).

Plasma CORT levels reflected a similar pattern as its upstream endocrine regulator, ACTH, yet there were striking differences in the response to EtOH between Ch- and E_2_-treated animals. Notably, in OVX animals that received E_2_ replacement there was a significant increase in plasma ACTH levels after an acute (one dose) EtOH exposure (p<0.001), yet a repeated binge-pattern of EtOH exposure did not increase plasma ACTH over saline controls (Fig. 3A, p = 0.15). Importantly, this effect was specific to E_2_-treatment, as there were no significant differences in plasma ACTH levels in acute, compared with repeated binge-pattern EtOH exposure, in the Ch-treated animals (Fig. 3A). In addition, CORT levels closely mirrored the effects of ACTH, as acute (p = 0.007), but not binge-pattern (p = 0.567), EtOH exposure significantly increased circulating CORT levels only in E_2_-treated animals (Fig. 3B). Finally, plasma CORT levels in the saline-treated group were significantly higher in E_2_-treated compared with Ch-treated OVX females (Fig. 3B, p = 0.032) as expected based on previous studies [11], [23], [24], [25].

### Effects of repeated binge-pattern EtOH exposure on CRH and AVP mRNA gene expression in Ch- or E_2_ – treated OVX adolescent female rats

**Figure 4:**
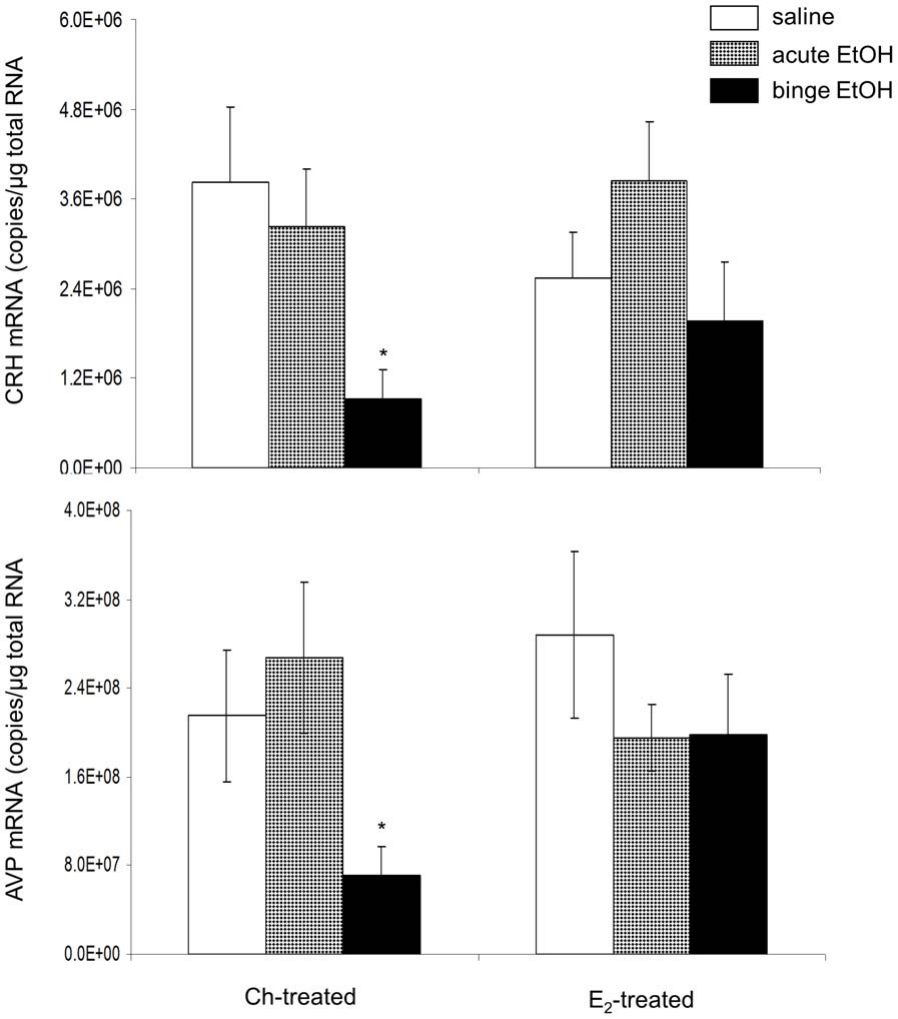
Effects of EtOH treatment on CRH and AVP gene expression in the PVN of ovariectomized female rats replaced with Ch or E_2_. CRH (A) and AVP (B) mRNA expression in the PVN of adolescent females replaced either with Ch or E_2_ and then treatment with either saline, acute, or binge EtOH. Data are expressed as mRNA copies/µg total RNA. (_*_) indicates a statistically significant difference from control (p<0.05).

CRH and AVP are upstream hypothalamic regulators of ACTH and CORT. Therefore, CRH and AVP mRNA were measured using qRT-PCR to determine the specific contribution of E_2_ on the female-specific stress responses to EtOH exposure. A two-way ANOVA revealed that there was a main effect of EtOH treatment on CRH and AVP mRNA expression and a statistically significant interaction between hormone and EtOH treatment on AVP mRNA expression. There were no main effects of hormone treatment on CRH (F(1,40) = 0.0575, p = 0.812) or AVP (F(1,40) = 1.008, p = 0.321) mRNA expression in the PVN Consistent with our previous studies and unlike what we have observed in male rats [11], neither acute, nor binge-pattern, EtOH exposure significantly altered CRH and AVP mRNA expression in the PVN of adolescent female rats given E_2_ replacement (Fig. 4). Unexpectedly however in OVX females that were not given E_2_ replacement, binge-pattern, but not acute EtOH exposure significantly decreased CRH (p = 0.03) (Fig. 4A) and AVP mRNA expression (p = 0.028, Fig. 4B), suggesting that E_2_ prevented binge-pattern EtOH-induced changes in CRH and AVP gene expression.

### 17β-Estradiol (E_2_) treatment in vitro abolished EtOH-induced changes in CRH promoter activity

**Figure 5:**
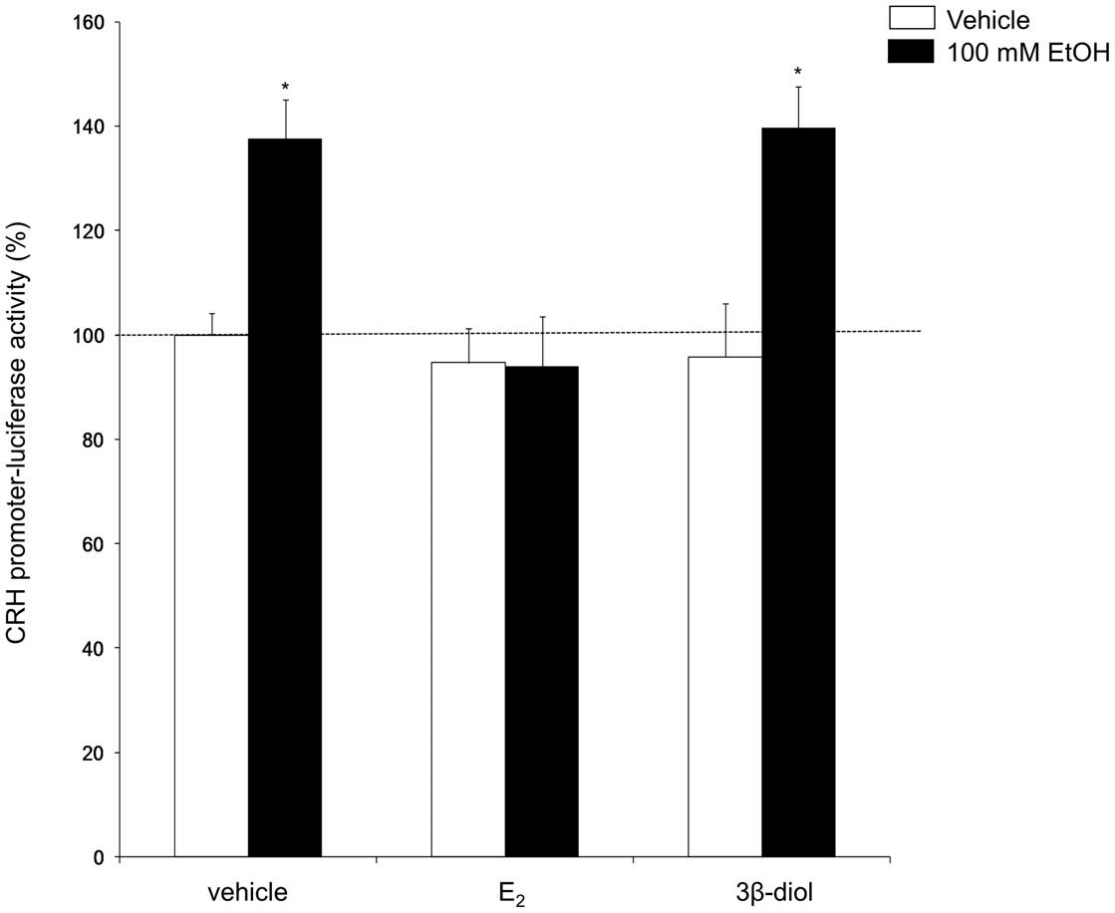
Effects of concomitant EtOH/E_2_ and EtOH/3βdiol treatment on CRH promoter activity in a neuronal cell line. *CRH promoter-l*uciferase activity was measured in IVB cells transfected with a CRH promoter-luciferase construct and treated with 100 mM EtOH for 2.0 h in media alone or in the presence of 10 nM E_2_ or 100 nM 3βdiol. Data are expressed as % change in luciferase activity of vehicle treated control. (_*_) indicates statistically significant difference compared to vehicle treated control (P<0.05).

The data shown in Figure 4 raised the possibility that E_2_ could prevent EtOH-induced alterations in CRH mRNA gene expression. To test possible interactions of EtOH with E_2_ on the activity of the CRH promoter, we transfected PVN-derived neuronal cells with a CRH promoter-luciferase construct and then co-treated with 100 mM EtOH in the presence or absence of 10 nM E_2_, or 100 nM of an ERβ specific agonist 3βdiol, and measured luciferase activity. Statistical analysis revealed that there was a main effect of alcohol (F(1,45) = 4.327, p = 0.043) and a main effect of hormone treatment (F(2,45) = 9.119, p<0.001) on CRH promoter activity. EtOH significantly increased CRH promoter activity as compared to vehicle-treated controls. (p<0.001) (Fig. 5) and this effect was abolished in the presence of 10 nM E_2_ (p = 0.947) (Fig. 5). Concomitant treatment with the ERβ specific agonist, 3β-diol, had no effect, suggesting the effects of E_2_ on CRH promoter activity are not mediated by ERβ.

### AVP mRNA expression in the SON after alcohol treatments in Ch- or E_2_–treated ovariectomized (OVX) females

**Figure 6:**
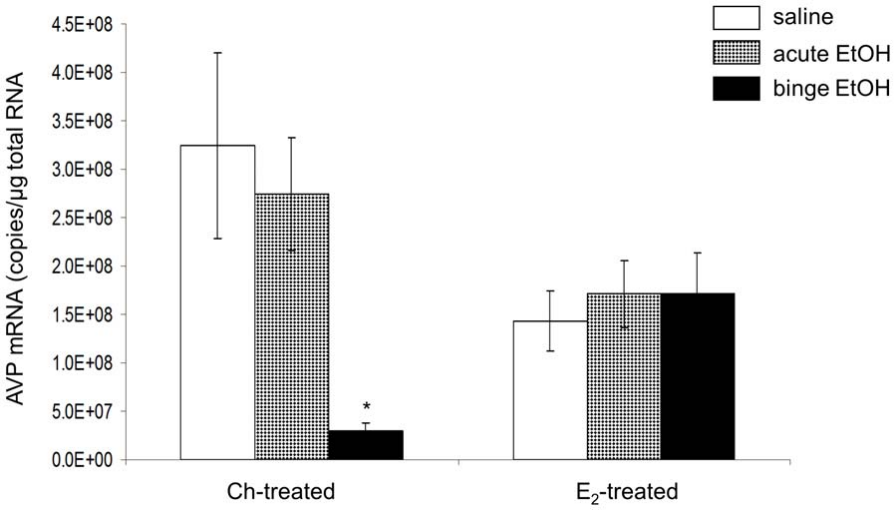
Effects of EtOH treatment on AVP gene expression in the SON of ovariectomized female rats. AVP mRNA expression in the SON of adolescent Ch- or E_2_ - treated female rats given saline, acute, or repeated binge-pattern EtOH. Data are expressed as mean ± SEM of AVP mRNA copies/µg total RNA. (_*_) indicates a statistically significant difference from control (p<0.05).

We have previously shown that in adolescent male and female rats EtOH did not change AVP mRNA expression in the SON, a main region responsible for osmoregulation, which indicated that the effects observed in the PVN were not due to the diuretic effects of EtOH. In this study, AVP mRNA expression in the SON was also measured in order to verify the HPA axis specificity of EtOH effects. Consistent with our previous observations in gonad intact animals, EtOH treatment did not induce any significant differences in SON AVP mRNA expression (p = 0.907) in E_2_-treated females, however binge-pattern EtOH treatment significantly decreased AVP mRNA levels in the Ch-treated group, as compared to saline controls (p<0.001) and acute EtOH treatment (p = 0.003). There were no differences between saline and acute EtOH groups within Ch-treated females (Fig. 6).

## Discussion

The key novel findings in this study demonstrate that the predominant female sex hormone, E_2_, was partly responsible for mediating the sex-specific effects of EtOH on the HPA axis, but it was not the only contributing factor. Previous work in our laboratory showed that repeated binge-pattern alcohol exposure in adolescent rats induced sexually dimorphic changes in the PVN expression of CRH and AVP, yet there were no sex differences observed in circulating plasma CORT levels, the downstream peripheral target of these genes [11]. These data suggested that EtOH dysregulated the HPA axis of adolescent rats in a sex specific manner, however the physiological mechanisms underlying these sexually dimorphic effects have not yet been fully described. Sex steroid hormones, specifically testosterone and/or E_2_, are logical contributors to the sex-specific responses in gonad intact rats. Indeed, the evidence provided in these studies point to a direct role for E_2_ in maintaining the reactive tone of the HPA axis in response to the physiological stressor, EtOH, and two clear mechanistic actions of E_2_ emerged: 1) at the level of the hypothalamus, whereby E_2_ treatment maintained normal steady state mRNA levels of CRH and AVP in the PVN in response to an EtOH stressor, and 2) in the reactivity of the HPA axis to a repeated stressor through E_2_-mediated modulation of the habituation response.

The evidence presented herein supports the hypothesis that, in females, the presence of E_2_ is required for the habituation of the HPA axis to repeated binge-pattern EtOH exposure. In E_2_ -treated females, repeated binge-pattern EtOH exposure failed to significantly increase plasma ACTH and CORT levels, which is consistent with HPA axis habituation to a repeated stressor. However, both acute and repeated binge-pattern EtOH exposure significantly increased circulating ACTH and CORT levels in Ch-treated controls and no habituation effect was observed. These data are also consistent with our previous report in which we showed that there was a habituation effect in gonad intact, adolescent, female rats following repeated binge-pattern EtOH exposure [11], [19]. Similarly, Lunga et al., [26] showed that OVX adult female rats had a diminished habituation response to a repeated restraint stress compared with OVX E_2_-replaced animals, further supporting a role for E_2_ in the female HPA axis habituation response. Interestingly, this becomes more complex in male rats, as a recent study by Bingham et al., [27] demonstrated that early postnatal activation of androgen receptors is also an important mediator of the adult habituation response to repeated stressors.

One of the most striking results in these studies showed that repeated binge-pattern EtOH exposure, but not acute, significantly decreased both AVP and CRH mRNA in OVX Ch-treated controls. This result was opposite from what we have observed in gonad intact males [11], where repeated binge-pattern EtOH exposure significantly increased CRH and AVP gene expression. It was also contrary to results from females, either intact or E_2_-replaced, where EtOH had no effect on CRH and AVP gene expression in the PVN. [28], [29], [30]. Taken together, these results suggest that E_2_ is required for maintaining the reactive tone of the HPA axis, possibly by stabilizing steady state CRH and AVP mRNA levels in females. It also raises the possibility that additional gonadally-derived factors might be required for mediating the EtOH-induced increase in CRH and AVP gene expression in males.

Steady state CRH mRNA is strongly correlated with CRH promoter activity. Stress-induced increases in circulating glucocorticoids inhibit CRH promoter activity, thereby facilitating a return of the HPA axis towards homeostatic balance [31], [32], [33]. We predicted that modulation of CRH promoter activity would be one possible mechanism for the observed E_2_-mediated maintenance of steady state CRH mRNA following EtOH exposure in females. Previous studies from our laboratory have shown that EtOH significantly increased CRH promoter activity after 2 hours of exposure at concentrations ranging from 12.5 to 100 mM [34]. Importantly for this study, concomitant treatment of EtOH and E_2_ abolished the effects of EtOH alone on CRH promoter activity (see Fig. 5), consistent with the observed lack of EtOH effect on CRH mRNA observed in both gonad-intact and E_2_-treated female rats. E_2_ is derived from the precursor hormone, testosterone (T), via conversion by the aromatase enzyme. However, T is also readily converted to 5α-dihydrotestosterone (DHT), which acts exclusively through androgen receptors, and DHT, can subsequently, be converted to 3βdiol [35], [36], [37]. Importantly, 3βdiol is a naturally occurring selective agonist for estrogen receptor β (ERβ) and has been shown to modulate AVP promoter activity and gene expression in the PVN [38], [39], [40], [41]. The EtOH-induced increase in CRH promoter activity was abolished in the presence of E_2_, but not 3βdiol, suggesting that the effects of E_2_ on CRH promoter activity are not mediated through ERβ. Moreover, these results establish that direct modulation of CRH promoter activity is a potential mechanism for E_2_-mediated maintenance of steady state CRH mRNA in the presence of EtOH.

Repeated binge-pattern EtOH exposure did not change AVP mRNA expression in the SON of E_2_ -replaced animals, yet it was significantly decreased in Ch-treated controls. Previous studies in our lab and others have shown that EtOH had no effect on AVP expression in the SON [28], or the magnocellular division of the PVN, in gonad intact female rats [30], which is consistent with our results from E_2_-replaced animals. Notably, however, we observed a significant decrease in AVP mRNA expression in the SON of Ch-treated controls. It is well accepted that E_2_ has neuroprotective effects after a neuronal insult [42], [43], [44], [45], [46], [47], [48], [49], and there is an increase in aromatase activity, the enzyme required for conversion of T to E_2_, after ischemic stroke and other brain injuries [47], [48], [49]. Therefore, one possibility is that OVX Ch-treated females lack E_2_-mediated neuroprotection and the population of CRH and AVP containing neurons in the PVN and SON are decreased as a result. Alternatively, alcohol exposure has also been shown to reduce AVP mRNA in the SON of rats bearing PVN lesions [28], suggesting that there is some cooperation between these two nuclei. In PVN-derived cells, AVP has been shown to be protective against serum starvation-induced cell death [50], [51]. Therefore, decreased AVP expression in the PVN, as was observed following repeated binge-pattern EtOH exposure in our Ch-treated controls, could have potentiated the reductions in AVP expression we observed in the SON.

Lastly, we showed that E_2_ -treated females had significantly lower body weights and gained weight at a slower rate as compared to Ch-treated females, but EtOH treatment had no effect on body weight. These findings are consistent with the known anorexigenic effects of E_2_, as lack of this hormone in humans, for example during the menopausal transition, tends to increase body weight [52]. In addition, body weight data in EtOH treated groups showed that the repeated binge-pattern alcohol exposure paradigm employed in our experiments did not inhibit the normal overall growth of the animals. This result was consistent with our previous data which showed no differences in body weight due to EtOH treatment in adolescent animals [11], [34]. In addition this binge EtOH exposure paradigm has been shown to be reliable for testing the effects of EtOH using an exposure pattern that is typical for adolescents [18].

Taken together, our data show for the first time that E_2_ plays a critical role in mediating the sexually dimorphic effects of repeated binge-pattern EtOH exposure during adolescence, and that these sex differences are mediated at the level of CRH promoter through the interplay of EtOH and estrogen receptor signaling.
